# Characterization of free fatty acid receptor family in rainbow trout (*Oncorhynchus mykiss*): towards a better understanding of their involvement in fatty acid signalisation

**DOI:** 10.1186/s12864-023-09181-z

**Published:** 2023-03-20

**Authors:** Jérôme Roy, Elodie Baranek, Lucie Marandel

**Affiliations:** INRAE, University of Pau and Pays de L’Adour, E2S UPPA, UMR1419 Nutrition Metabolism and Aquaculture, Aquapole, F-64310 Saint-Pee-Sur-Nivelle, France

**Keywords:** G protein-coupled receptors, Synteny, Phylogenetic tree, Development, Feeding

## Abstract

Since 20 years of research, free fatty acids receptors (FFARs) have received considerable attention in mammals. To date, four FFARs (FFAR1, FFAR2, FFAR3 and FFAR4) are especially studied owing to their physiological importance in various biological processes. This ubiquitist group of G protein-coupled receptors (GPCRs) are majors reports in the key physiological functions such as the regulation of energy balance, metabolism or fatty acid sensing. However, up till date, even some studies were interested in their potential involvement in fatty acid metabolism, no genome investigation of these FFARs have been carried out in teleost fish. Through genome mining and phylogenetic analysis, we identified and characterised 7 coding sequences for *ffar2* in rainbow trout whereas no *ffar3* nor *ffar4* gene have been found. This larger repertoire of *ffar2* genes in rainbow trout results from successive additional whole-genome duplications which occurred in early teleosts and salmonids, respectively. A syntenic analysis was used to assign a new nomenclature to the salmonid *ffar2* and showed a clear conservation of genomic organisation, further supporting the identity of these genes as *ffar2*. RT-qPCR was then used to examine, firstly during ontogenesis and secondly on feeding response the expression pattern of *ffar1* and *ffar2* genes in proximal gut and brain of all trout *ffar* genes. Overall, this study presents a comprehensive overview of the *ffar* family in rainbow trout.

**Supplementary Information:**

The online version contains supplementary material available at 10.1186/s12864-023-09181-z.

## Introduction

In addition to act as energy sources, free fatty acids (FFAs) are essential nutrients that contribute to various cellular functions and exert biological effects through several signaling pathways [1]. In order to detect FFAs, the free fatty acid receptors (FFAR), a specific family of G protein coupled receptors (GPCRs), has been identified as the main receptors mediate effects of different FFAs [2]. The successful sequencing of the human genome has greatly accelerated the researches in this domain. Indeed, among various research endeavors benefiting from established genomic information, one of the most fruitful areas is the research on GPCRs. Thus, human FFARs sequences were originally identified as a cluster of four intronless genes located at locus 19q13.1 [3]. This discovery has subsequently allowed the emergence of researches on their functions, their ligands and their regulations [4]. Even if more than 800 types of GPCRs are reported in the human genome, only few of them have been identified and characterized in mammals to be activated by FFAs of various chain lengths [5]. To date, four FFARs have received considerable attention owing to their physiological importance in various biological processes, such as facilitation of insulin and incretin hormone secretion, adipocyte differentiation, anti-inflammatory effects, neuronal responses at least in mammals [6]. In particular, FFAR1 (GPR40) and FFAR4 (GPR120) are activated by long-chain (LC) saturated and unsaturated fatty acids, while FFAR3 (GPR41) and FFAR2 (GPR43) are activated by short chain fatty acids (SCFAs), mainly acetate, butyrate, and propionate [6]. FFARs as GPCRs are widely expressed in various tissues and contribute to many important physiological functions that maintain energy balance and immune homeostasis. Also, among their physiological importance in mammals, FFARs play a crucial role in fatty acid sensing and their regulation especially in brain and gut [7, 8].

In the farmed fish sector, the ongoing expansion of aquaculture enforces an urgent need to find alternative ingredients to replace the use of the traditional ingredients in aquafeed composition, meaning fish meal and fish oil (FM/FO). However, for rainbow trout (*Oncorhynchus mikyss*), one of the most produced species in Europe, despite 20 years of research [9], the total replacement of marine products by plant ingredients has still not been achieved due to the drastic alteration of survival rate and growth performance [10] which could be mainly related to alteration in the regulation of feeding behaviour and feeding efficiency. Indeed, a disadvantage of using plant ingredients is the modification of the nutrient composition of the diet, especially fatty acid composition. By far, the major difference in terms of nutrient replacement in plant-based diets *versus* commercial diets (containing FM/FO) is the lack of ω-3 long chain polyunsaturated fatty acids (ω-3 LC-PUFA), mainly eicosapentaenoic acid (EPA) and docosahexaenoic acid (DHA) [11]. Some studies reported that this absence of ω-3 LC-PUFA in plant products, especially DHA, is known to affect feeding behavior during the whole fish life-cycle by reducing feed intake [12] and feeding rhythms [13], and by increasing abnormal feeding behavior [14]. Recently, we revealed that rainbow trout have the fundamental mechanisms (sensory receptors) for nutrient perception related to different diet composition enriched or not with ω-3 LC-PUFA [15, 16] and that ω-3 LC-PUFA controlled its feeding behavior [17]. Furthermore, we observed that a diet rich in ω-3 LC-PUFA impacted in a relatively high proportion the brain function [18] and brain lipid content [19] in rainbow trout. These recent studies disclosed implications of ω-3 LC-PUFA in the modulation and regulation of the control of feeding behaviour particularly the FA sensing pathways.

Thus, the knowledge on fat detection sensing mechanisms by FFARs in fish and their implication in metabolism regulation is still in its infancy. Especially, in farmed fish, the understanding of the mechanisms regulating feed intake remains a major challenge to be studied for the development and maintenance of a sustainable aquaculture. To achieve optimal animal growth, the development of aquaculture have to rely now on the production of cost-effective and nutritionally adequate aquafeed. It is thus essential to improve fish feeding strategies (optimizing food consumption), by reducing economic losses due to non-ingested food and in the same time reducing the part of FM/FO in the composition of aquafeed.

Firstly, identification and characterization of *ffar* genes can be crucial to better understand nutritional sensing processes in salmonids. Yet, despite the rapid accumulation of genomic and transcriptomic information in teleost fish, coding sequences for *ffar* have not been widely characterized yet. Moreover, during vertebrates’ evolution several rounds of whole genome duplication (WGD) occured [20], the first one at the emergence of chondrichthyes, the second at the radiation of teleosts and the teleost-specific genome duplication (3R; TGD). Then an additional duplication occured in the salmonids; Salmonid specific genome duplication, SaGD. These WGD duplications led to genes’ duplications, leading to adaptive innovation via the conservation of duplicated genes available for the evolution of new functions [20]. Thus, to assess more comprehensively the functional importance of *ffar* in rainbow trout, it seems essential to firstly decipher and characterize them within the trout genome by phylogenetic and syntenic analysis. Secondly, to understand how the identified *ffar* genes could lead to or at least be one factor involved in the fatty acid perception and their regulation in rainbow trout, it is important to consider their transcriptional behaviour during ontogenic period knowing to be a window of metabolic plasticity and during feeding period to observe their regulation by diet changes.

The aim of the present study was thus to: (i) identify and propose new nomenclature of the *ffar* genes in rainbow trout by phylogenetic and syntenic analysis; (ii) determine their expression pattern at critical developmental stages and in juvenile trout before and after nutritional challenge by commercial-like diet or by plant-based diet in two mains organs involved in the regulation of energy balance and fatty acid metabolism, *i.e.* the gut and the brain tissue.

## Materials and methods

### Ethics statement

The animal study was reviewed and approved by French National Consultative Ethics Committee. The study was conducted according to the guidelines of the National Legislation on Animal Care of the French Ministry of Research (Decree No 2013–118, 1 February 2013) and in accordance with EU legal frameworks relating to the protection of animals used for scientific purposes (*i.e.* Directive 2010/63/EU). The experiment was conducted at the INRAE NuMeA facilities (https://doi.org/10.15454/GPYD-AM38), and approved by the ethical committee (C2EA-73) of INRAE “Comité d’éthique Aquitain poissons oiseaux (Aquitaine Fish and Bird Ethics Committee)” (agreement number INRAE 21,699, 19^th^ December, 2019). The scientists in charge of the experiments received training and personal authorization.

### Experimental diets design

Diets were manufactured at INRAE experimental facilities at Donzacq using a twinscrew extruder (Clextral). Pellets size were produced between 4 mm diameter and 4 mm length. Details about the ingredients and composition of the experimental diets are given in Table 1 and the proportions of the main FA in the diets in Table 2. The experiment was conducted with one of the two different experimental diets: a control diet containing a mix of FM (19%), FO (8%) and plant ingredients, and a plant-based diet, completely free from FM and FO, which were replaced by a blend of plant ingredients (8% of rapeseed oil, 6% of linseed oil and 3.6% palm oil). This vegetal oil blend in plant-based diet was chosen in order to provide an overall amount of FA classes in proportion similar to those of FA classes found in control diet. For plant-based diet diet, DHA and EPA (present in FO for control diet) was replaced to the benefit of alpha-linolenic acid (ALA) by adding linseed oil (6%).

**Table 1:** Selected fatty acid composition (% total fatty acids)

	Diet	
	**Commercial-like diet**	**Plant-based diet**
C12:0	0.08	0.22
C14:0	3.76	0.65
C15:0	0.23	0.05
C16:0	12.78	13.65
C17:0	0.23	0.09
C18:0	2.76	2.81
C20:0	0.35	0.34
C22:0	0.22	0.19
C24:0	0.04	0.09
Sum of saturated fatty acids	**20.45**	**18.09**
C14:1 ω-7	0.09	0.0
C16:1 ω-7	3.76	0.55
C17:1 ω-7	0.1	0.04
C18:1 ω-9	33.06	38.55
C20:1 ω-9	1.3	0.61
C22:1 ω-9	0.9	0.12
Sum of MUFAs	**39.20**	**39.87**
C16:2 ω-4	0.76	0.02
C16:3 ω-4	0.83	0.12
C18:2 ω-4	0.19	0.02
C18:3 ω-4	0.1	0.08
Sum of ω-4 PUFAs	**1.88**	**0.24**
C18:2 ω-6 (LA)	15.86	21.23
C18:3 ω-6	0.14	0.0
C20:2 ω-6	0.10	0.05
C20:3 ω-6	0.06	0.0
C20:4 ω-6 (ARA)	0.48	0.0
C22:2 ω-6	0.0	0.08
C22:4 ω-6	0.0	0.0
C22:5 ω-6	0.12	0.0
Sum of ω-6 LC-PUFAs	**16.76**	**21.36**
C16:4 ω-3	0.0	0.0
C18:3 ω-3 (ALA)	4.96	18.75
C18:4 ω-3	0.93	0.09
C20:3 ω-3	0.0	0.0
C20:4 ω-3	0.3	0.0
C20:5 ω-3 (EPA)	7.94	0.77
C21:5 ω-3	0.32	0.05
C22:5 ω-3	0.96	0.08
C22:6 ω-3 (DHA)	4.06	0.39
Sum of ω-3 LC-PUFAs	***19.47***	***20.13***
Sum of ω-3 (EPA + DHA)	***12.0***	***1.16***
ω-3 (DHA + EPA) / ω-6	***0.72***	***0.05***

**Table 2:** Nucleotide sequence of the PCR primers used to evaluate mRNA expression of FFAR transcripts by RT-qPCR

**Transcript**	**Forward Primer**	**Reverse Primer**	**Database**	**Accession Number**
**Reference**				
*eef1a1*	**TCCTCTTGGTCGTTTCGCTG**	**ACCCGAGGGACATCCTGTG**	**Ensembl**	**ENSOMYG00000038328**
**FFARs**				
*ffar1*	**ACTGTTGCACCTGAGTCTGG**	**GCTGGTCCTGGGTGAAGTTC**	**Ensembl**	**ENSOMYG00000041396**
*ffar2a1a*	**CCGAGTTCCTCTGCTCCATC**	**TAGGTGATGGGGAAGGCAAC**	**Ensembl**	**ENSOMYG00000004986**
*ffar2a2*	**GACAACTTCACCCAGGAGCA**	**AGCAGAACACACAGGCCAG**	**Ensembl**	**ENSOMYG00000030315**
*ffar2b1.1*	**TTTTCCACACACAGTTGGCC**	**AGGTAGTGTTGTCGGCATCT**	**Ensembl**	**ENSOMYG00000041393**
*ffar2b1.2*	**GTGTGGCCTTCCCTATCAGA**	**GCAGGGCACAATGTACACAA**	**Ensembl**	**ENSOMYG00000041387**
*ffar2b2a*	**CCCATCCAACACTCGCTGAA**	**TGATGACGACGATGCTCAGG**	**Ensembl**	**ENSOMYG00000030493**
*ffar2b2b1*	**TGACCGCAATCAGTGTCGAA**	**CCCAGAAGAAGACGCTAGCC**	**Ensembl**	**ENSOMYG00000030500**
*ffar2b2b2*	**GTCCAGTACCATCAACGCCA**	**CTGCACACTCTCCAACAGGGT**	**Ensembl**	**ENSOMYG00000005604**

The two experimental diets contained 23.6% for commercial-like diet and 20.35% for plant-based diet of crude lipids with the same amount of major *ω*-3 FA; 19.47% in commercial-like diet and 20.13% in plant-based diet. This amount of *ω*-3 FA class was chosen in order to be close to the proportions of *ω*-3 FA classes found in marine diet [10]. In order to avoid exceeding anti-nutrient threshold levels, we used a blend of wheat gluten, soybean meal and whole wheat, corn gluten meal, soy protein and fatabean as protein sources (*c.* 46.11% of total diet). Synthetic L-lysine, L-methionine, dicalciumphosphate and soy-lecithin were added to all diets to correct the deficiency in essential amino acids, phosphorous and phospholipids. Mineral and vitamin premix were added to each diet. Diets were isoenergetic (*c.* 24.41 kJg^-1^ of dry diet) and were formulated to cover the nutritional requirements of the rainbow trout [21]. Nutrient compositions of the diets, crude protein and lipids, gross energy, ash and starch content and fatty acid profils were analyzed as previously described [16].

### Experimental design

For ontogenesis analysis, oocytes were fertilized synchronously with neomale sperm and reared in separate tanks at 8 °C in our experimental facilities (INRAE Fish Farm of Lees-Athas, Permit number A64.104.1, vallée d’Aspe, France) as previously described [22]. Rainbow trout were sampled according to spawn origin before fertilization (oocyte) and then during development according to Vernier [23] at stages 5, 6, 7, 8, 10, 12, 15, 22 and 23. Embryos were directly snap-frozen, whereas alevins were killed by terminal anaesthetization by bathing in benzocaine prior to pooling and storage in liquid nitrogen. The samples were stored at - 80 °C until mRNA analysis.

For nutritional analysis, at the beginning of the experiment, female rainbow trout fry come from the same parental stock (INRAE Fish Farm of Lees-Athas, Permit number A64.104.1, vallée d’Aspe, France). The feeding experiment was conducted in a recirculating rearing system at the INRAE facilities of Donzacq, France (authorisation number A40-228.1, Landes). At the beginning of the experiment, female rainbow trout with mean weight of 140 g were randomly distributed among 6 tanks of 100L (50 fish per tank). Water flow was set to ensure an oxygen concentration above 90% saturation. Fish were exposed to natural photoperiod condition and the water temperature was set at 15 ± 1 °C. During the trial, water dissolved oxygen was 9 mg L^-1^, ammonia < 0.01 mg L^-1^, nitrite < 0.04 mg L^-1^, nitrate was about 17 ppm. The quantity of flow was 0.3 l/s by tank, all water of each tank was changed 6 times each hour. During 30 days, all fish were fed by hand twice a day with an interval 8 h with commercial-like diet, until apparent satiety. After 30 days, 5 days of fasted period was realized. After that, trout were fed a single meal with commercial-like diet for three tanks and plant-based diet (Diet composition Table 3) for the three other tank. For sampling, fish was killed by terminal anesthetization by bathing in benzocaine (30 mg/l then a bath at 60 mg/l) and proximal gut and whole brain were sampled before the single meal (T = 0), 20mns after the single meal (T = 0.2), 4 h after the single meal (T = 4) and 24 h after the the single meal (T = 24). Tissues were then frozen in liquid nitrogen and stored at - 80 °C until mRNA analysis.

**Table 3:** Ingredients and composition of the experimental diets

Ingredient (%)	DIET	
	**Commercial-like diet**	**Plant-based diet**
Fish meal	19.0	0.0
Soybean meal	11.0	18.0
Extruded whole wheat	13.5	11.5
Corn gluten	6.0	15.2
Wheat gluten	0.0	8.0
Soy protein concentrate	18.0	6.8
Fababean protein concentrate	9.5	8.0
Soy lecithin	2.5	2.5
L-Lysine	0.6	7.16
L-methionine	0.6	0.6
CaHPO4.2H2O	0.1	1.44
Mineral premixa	1.4	2.0
Vitamin premixb	1.3	1.2
Fish oil	8.0	0.0
Rapeseed oil	8.5	8.0
Palm oil	0.0	3.6
Linseed oil	0.0	6.0
Composition (% of dry matter)		
Dry matter (in % of diet)	97.60	94.83
Crude protein	45.28	46.95
Crude lipid	23.6	20.35
Starch	17.66	11.37
Ash	4.10	7.58
Energy (kJg-1 DM)	24.49	24.33

^aMineral^ premix: (g or mg kg - 1 diet): calcium carbonate (40% Ca), 2.15 g; magnesium oxide (60% Mg), 1.24 g; ferric citrate, 0.2 g; potassium iodide (75% I), 0.4 mg; zinc sulphate (36% Zn), 0.4 g; copper sulphate (25% Cu), 0.3 g; manganese sulphate (33% Mn), 0.3 g; dibasic calcium phosphate (20% Ca, 18% P), 5 g; cobalt sulphate, 2 mg; sodium selenite (30% Se), 3 mg; KCl, 0.9 g; NaCl, 0.4 g (UPAE, INRA)

^bVitamin^ premix: (IU or mg kg - 1 diet): DL-a tocopherol acetate, 60 IU; sodium menadione bisulphate, 5 mg; retinyl acetate, 15,000 IU; DL-cholecalciferol, 3000 IU; thiamin, 15 mg; riboflavin, 30 mg; pyridoxine, 15 mg; B12, 0.05 mg; nicotinic acid, 175 mg; folic acid, 500 mg; inositol, 1000 mg; biotin, 2.5 mg; calcium pantothenate, 50 mg; choline chloride, 2000 mg (UPAE, INRA)

### In silico analysis

*Ffars* genes and related protein sequences were identified in the Genomicus software program, version 106.01, 2021–08-15 (https://www.genomicus.bio.ens.psl.eu/genomicus-106.01/cgi-bin/search.pl) and collected from Ensembl (http://www.ensembl.org, Ensembl Release 102; November 2020, RT genome available). The genoscope database (http://www.genoscope.cns.fr/ trout) was used to identify *ffar* related genes in the rainbow trout genome using BLAST analysis. Sequences are available under the accession numbers reported in Table 3. Ensembl database (http://www.ensembl.org/index.html) was also used to collect amino acids deduced sequences of *ffar* for all species studied.

Protein alignment and the percentage Identity Matrix established with amino acids deduced sequences were performed using MUSCLE software (https://www.ebi.ac.uk/Tools/msa/muscle/). The Protein sequence encoded by FFAR of trout are presented in supplemental information.

The evolutionary history of the four *ffar* was inferred using the Neighbor-Joining method [24]. The optimal phylogenetic tree is shown. The percentage of replicate trees in which the associated taxa clustered together in the bootstrap test (500 replicates) are shown next to the branches [25]. The tree is drawn to scale, with branch lengths in the same units as those of the evolutionary distances used to infer the phylogenetic tree. The evolutionary distances were computed using the Poisson correction method [26] and are in the units of the number of amino acid substitutions per site. This analysis involved 57 amino acid sequences. All ambiguous positions were removed for each sequence pair (pairwise deletion option). There were a total of 530 positions in the final dataset. Evolutionary analyses were conducted in MEGA11 [27].

Gene synteny analysis was carried out for all *ffar* genes between salmonid and other relevant genomes using Genomicus software. To determine the genomic neighbourhood around candidate genes and the conservation of gene order across species, genes were visually examined in NCBI’s genomic region browser and Ensembl Gene Summary databases. The phylogenetic analysis showed that all the gar and teleosts sequences rooted together with FFAR2 tetrapods sequences and were divided in 2 distinct sub-trees that we respectively named *ffar2a* and *ffar2b*.

### mRNA levels measurement by real-time quantitative PCR

Total RNA was extracted from the oocytes and embryos (*N* = 3 pools), proximal gut and brain, (*N* = 6 per fish) using the TRIzol reagent method (Invitrogen, Carlsbad, CA) with Precellys®24 (Bertin technologies, Montigny le Bretonneux, France) following Trizol manufacturer’s instructions. Luciferase control RNA (Promega), 10 pg per 1.9 mg of embryo/alevin or oocyte, was added to each sample for ontogenesis analysis to allow for data normalization during early development as previously described [22, 28]. Total RNA (2 µg) was used for cDNA synthesis. RNA purity was tested by optical density (OD) absorption ratio (OD 260 nm/280 nm) using a NanoDrop 2000c (Thermo, Vantaa, Finland), and only samples with an OD 260 nm/280 nm ratio > 1.8 were used for analysis. The Super-Script III RNAse H-Reverse transcriptase kit (Invitrogen) was used with random primers (Promega, Chartonniéres-les-bains, France) to synthesize cDNA in a final volume reaction of 20 µl, according to the manufacturer’s instructions. QPCR assays were performed with the Roche Lightcycler 480 system (Roche Diagnostics, Neuilly-sur-Seine, France). The reaction mix was 6 µl per sample, including 2 µl of diluted cDNA template (1:10), 0.24 µl of each primer (10 µmol l^-1^), 3 µl of Light Cycler 480 SYBR® Green I Master mix and 0.52 µl of DNAse/RNAse-free water (5 Prime GmbH, Hamburg, Germany). The qPCR protocol was initiated at 95 °C for 10 min for the initial denaturation of the cDNA and hot-start Taq-polymerase activation, followed by 45 cycles of a two-step amplification program (15 s at 95 °C; 10 s at 60 °C) [16]. Cycle thresholds values superior than 35 cycles were not considered. Melting curves were monitored systematically (temperature gradient 0.11 °C per second from 65 to 97 °C) at the end of the last amplification cycle to confirm the specificity of the amplification reaction. Duplicate wells were used for each sample and negative controls were included in all reactions, consisting in wells containing RNA samples and water instead of cDNA. Efficiency of all qPCR reactions was 96–100% and R2 was 0.95–1. For ontogenic analysis, data were subsequently normalized to the exogenous luciferase transcript as previously described [22]. The different PCR products were initially checked by sequencing to confirm the nature of the amplicon. For this, primers were tested on a pool of cDNA and amplified PCR products were sequenced. Primer sequences and accession numbers are presented in Table 2. For nutritional analysis, data from mRNA gut and brain were extrapolated from standard curves and normalized to the housekeeping gene after validation; elongation factor 1a gene (*eef1a*). Relative expression of the target genes was determined by the **ΔΔ**CT method [29]. mRNA sequences of trout found and used in this study are available in NCBI and Ensembl genome browser.

### Statistical analysis

All statistical analyses were performed using R software (version 3.6.1, R development Core Team, 2008)/Commander package. Data are presented as mean ± standard error of the mean (SEM). Analyses were carried out on untransformed data as criteria for normality and homogeneity of variances were fulfilled (Shapiro–Wilk’s and Levene’s test, respectively). If the criteria (normality and homogeneity) were still not met, a non-parametric test was used for the analysis. Diet effect and time effect were analyzed using two-way ANOVA. If interaction was detected (*p-value* <0.05), data were finally analyzed using one-way ANOVA to test the diet effect and time effect individually. A Tukey’s was used as a post hoc test (*p-value* < 0.05).

## Results

### Phylogenetic analysis of *ffar1*

**Fig. 1:**
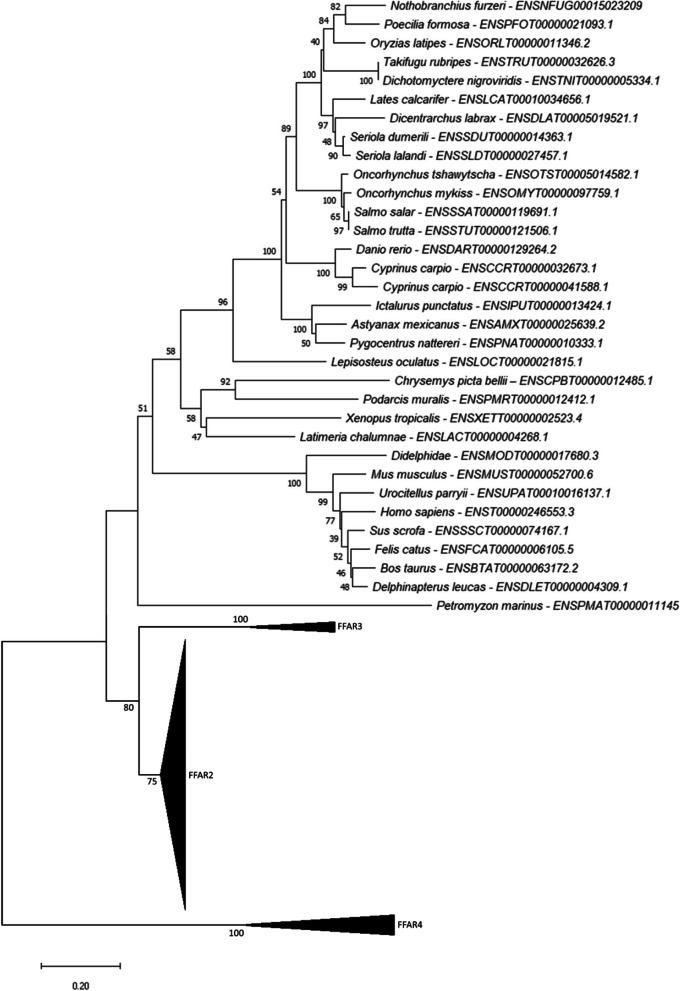
Phylogenetic tree of *ffar1* in vertebrates. FFAR1 protein sequences were aligned using the Maximum Likelihood Method (with Poisson model). Following alignment, the phylogenetic tree was constructed using the neighbour-joining method in Molecular Evolutionary Genetics Analysis (MEGA) software version 7.0 (Tamura 2013). The branch support values were gained by non-parametric bootstrapping (500 replicates). The scale bar represents the calculated evolutionary distance. Genbank accession numbers (from Ensembl or Genoscope databases) are specifed for each species. Mammalian and teleost FFAR2 protein sequences were used to root the tree

By analysing key vertebrate genomes available in Ensembl (Release 102; November 2020), we found one gene related to the sarcopterygii* ffar1* in the spotted gar as well as in the analysed teleosts (except for common carp with two encoding genes) including salmonids (Fig. 1). Our phylogenetic analysis showed that both gar and teleost sequences grouped together with tetrapods Ffar1 protein sequences and more generally with actinopterygii selected sequences.

### Phylogenetic and syntenic analysis of *ffar2*

We first collected annotated Ffar2 amino acids sequences in selected tetrapod species in Ensembl. We found only one annotated sequence for each selected species (*i.e.* mouse, rat, human, pig, chicken). Using the Blast function in Ensembl and Genomicus software V.106.01, we collected tetrapod-Ffar2 related sequences in selected teleosts species including 3 salmonids (*i.e.* huchen, atlantic salmon, rainbow trout), species selected to cover the major orders of teleosts. We identified three amino acids sequences related to tetrapods Ffar2 in the spotted gar genome, and between three and nine sequences in teleosts (4 for the zebrafish (*Danio rerio*), 6 for the Mexican tetra (*Astyanax mexicanus*), 4 for the Nile tilapia (*Oreochromis niloticus*), 3 for the Japanese medaka (*Oryzias latipes*), 3 for the fugu (*Takifugu*), 9 for the huchen (*hucho hucho*), 8 for the atlantic salmon (*salmo salar*) and 7 for rainbow trout).

**Fig. 2:**
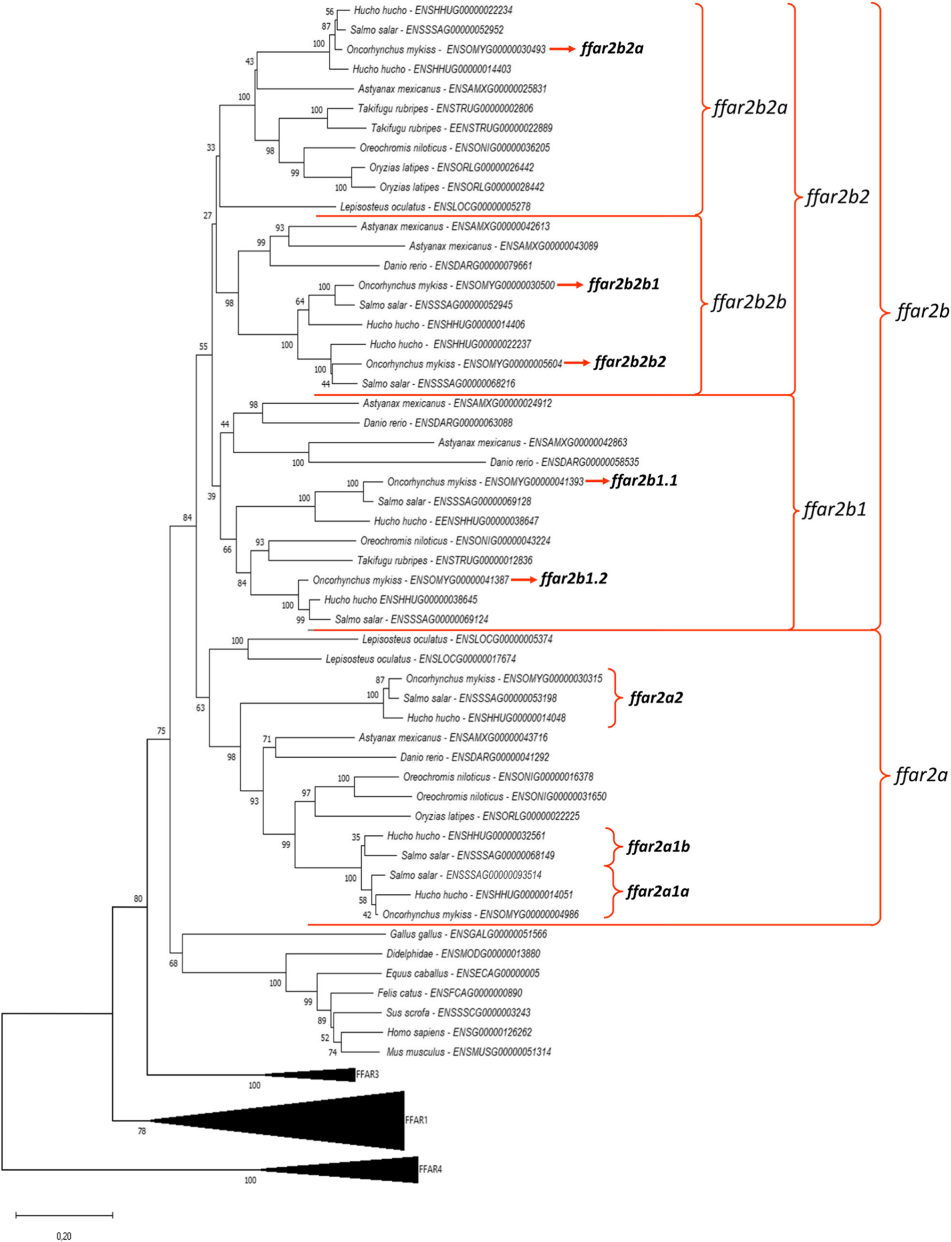
Phylogenetic tree of *ffar2* in vertebrates. FFAR2 protein sequences were aligned using the Maximum Likelihood Method (with Poisson model). Following alignment, the phylogenetic tree was constructed using the neighbour-joining method in Molecular Evolutionary Genetics Analysis (MEGA) software version 7.0 (Tamura 2013). The branch support values were gained by non-parametric bootstrapping (500 replicates). The scale bar represents the calculated evolutionary distance. Genbank accession numbers (from Ensembl or Genoscope databases) are specifed for each species. Mammalian and teleost FFAR2 protein sequences were used to root the tree

**Fig. 3:**
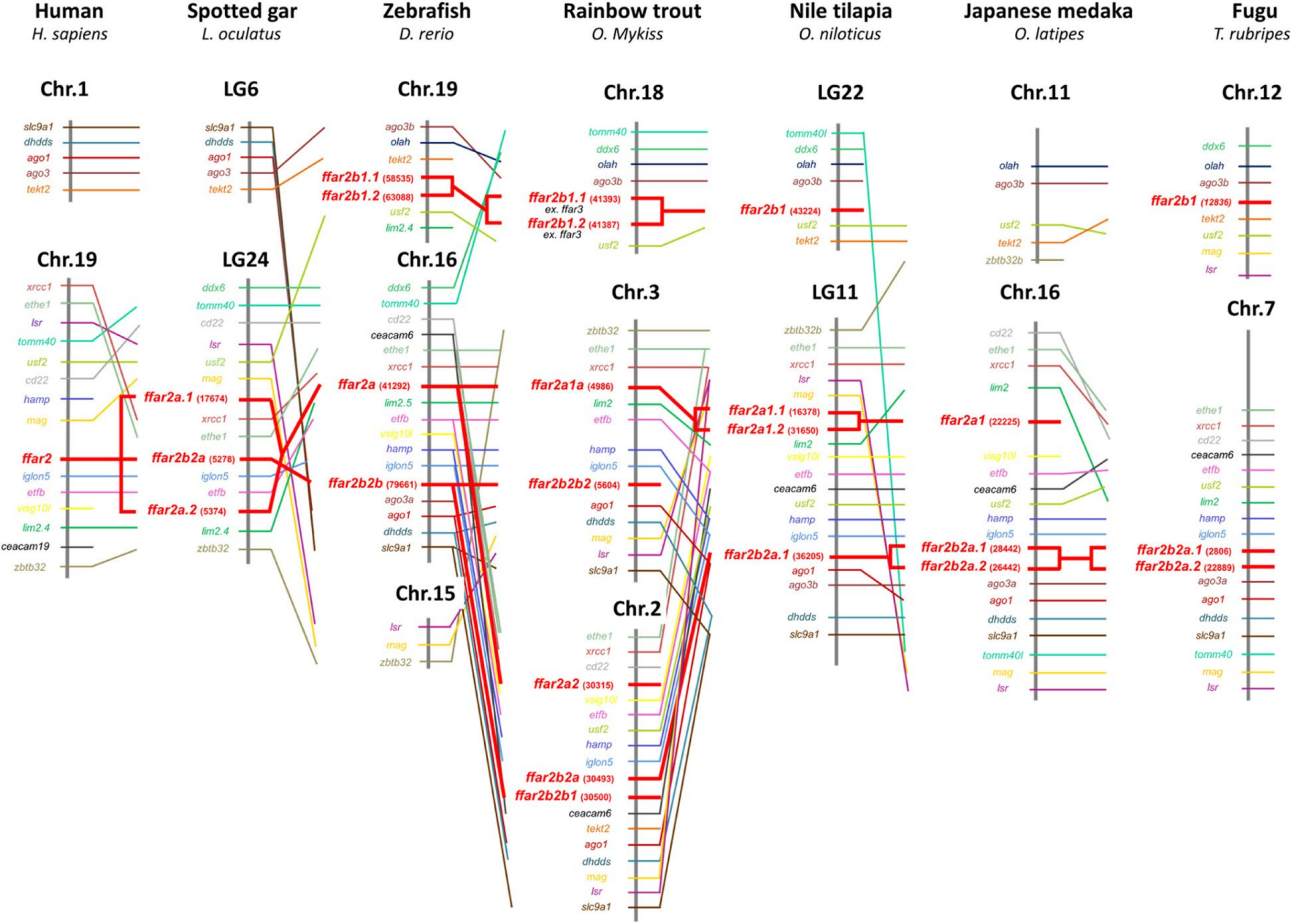
*ffar2* gene synteny in selected vertebrates. The syntenically conserved gene blocks are shown in matching colours. Gene synteny was compiled from up- and down-stream locations relative to each species *ffar2* taken from NCBI’s genome browser and using Genomicus software (https://www.genomicus.bio.ens.psl.eu/genomicus-100.01/cgi-bin/search.pl). Species names are displayed at the top of the figure, chromosome number and range (position) are shown above each species gene synteny. Chr., chromosome; LG., liguleless gene

Regarding the *ffar2a* sub-tree (Fig. 2), the sequence ENSOMYG00000004986 from the rainbow trout grouped together with other teleosts sequences whereas a subtree was composed of the trout sequence ENSOMYG00000030315 grouping together with additonnal salmonid sequences (*i.e.* sequences ENSSAG00000053198 in salmon and ENSHHUG00000014048 in huchen). We named these two sequences *ffar2a1a* and *ffar2a2*, respectively. This tree configuration is in favour of a *ffar2a* duplication occurring before or around the teleost radiation with a probable loss of one copy of the gene in non-salmonid species. Such hypothesis seemed to be confirmed by our synthenic analysis (Fig. 3) showing that the syntenic group *usf2*-*ffar2a*-*ffar2b*-*lim2.4* containing both *ffar2a* and *ffar2b* in the spotted gar on chromosome LG24 is found duplicated in zebrafish on chromosome 16 and 19 but with a loss of *ffar2a* loci on the lastest chromosome. In addition, the unique trout *ffar2a1a* sequence grouped together in the phylogenetic tree with 2 sequences in salmon and 2 in huchen (ENSSAG00000068149, ENSSAG00000093514 and ENSHHUG00000032561, ENSHHUG00000014051, respectively) which were included in the same synthenic group (*hamp-usf2-etfb-lim2-ffar2a-xrcc1-ethe1*) found duplicated on 2 distincts chromosomes in these 2 species (ssa02 and ssa05 in salmon and QNTS01001724.1 and QNTS01000300.1 in huchen; data not shown).

The sub-tree that we named ffar2b, was in turn divided into 2 main subtrees respectively called *ffar2b1* and *ffar2b2* containing respectively 2 (accession number ENSOMYG000000041393 and ENSOMYG000000041387) and 3 (accessions numbers ENSOMYG00000030493, ENSOMYG00000030500, ENSOMYG00000005604) rainbow trout sequences.

In the ffar2b1 subtree, we named these two sequences *ffar2b1.1* and *ffar2b1.2*, respectively.

These two sequences for zebrafish (ENSDARG000000063088 and ENSDARG000000058535) and mexican tetra (ENSAMXG000000042863 and ENSAMXG000000024912) grouped together. The sequence ENSOMYG000000041393 (*ffar2b1.1*) from the rainbow trout grouped together only with salmonids sequences (*i.e.* sequences ENSSAG00000069128 in salmon and ENSHHUG00000038647 in huchen). The sequence ENSOMYG000000041387 (*ffar2b1.2*) from the rainbow trout grouped together with others teleost sequences (*i.e.* sequences ENSONIG00000043224 in nile tilapia, ENSTRUG00000012836 in fugu, ENSSAG00000069124 in salmon and ENSHHUG00000038645 in huchen). This tree configuration is in a favour of a *ffar2b1* tandem duplication *ffar2b1* (into *b1.1*, *b1.2*) resulting of the duplication in salmonids.

The syntenic analysis (Fig. 3) seemed to confirm this hypothesis showing that this two sequences *ffar2b1.1* and *ffar2b1.2* were included in the same synthenic group (*ago3-olah-tekt2-ffar2b1-usf2-lim2.4*) for zebrafish and mexican tetra (data not shown) and same synthenic group (*tomm40-ddx6-olah-ago3-ffar2b1-usf2*) for rainbow trout, atlantic salmon, huchen (data not shown for salmon and huchen), nile tilapia and fugu. *ffar2b1* were found duplicated on same chromosome in this syntenic group for zebrafish (*ffar2b1.1* and *ffar2b1.2*, Chr.19), mexican tetra (APWO02000060.1), rainbow trout (Chr.18), atlantic salmon (ssa27), huchen (QNTS01000383.1; data not shown for salmon and huchen) but with one copy for nile tilapia (LG.22) and fugu (Chr12).

Considering the ffar2b2 subtree, the three sequences were in turn divided into 2 others subtrees respectively called *ffar2b2a* containing respectively 1 (ENSOMYG000000030493) rainbow trout sequence and *ffar2b2b* containing respectively 2 (accessions numbers ENSOMYG000000030493) rainbow trout sequences (accessions numbers ENSOMYG00000030500, ENSOMYG00000005604) and named *ffar2b2b1* and *ffar2b2b2* respectively. The sequences ENSOMYG000000030493 and ENSOMYG000000030500 from the rainbow trout grouped together with two other teleosts sequences respectively (*i.e.* sequences ENSSAG00000052952 and ENSSAG00000052945 in salmon and ENSHHUG00000022234 and ENSHHUG00000014406 in huchen) and with zebrafish (ENSDARG000000079661), Mexican tetra (ENSAMXG000000042613 and ENSAMXG000000025831). This tree configuration is in a favour of a *ffar2b2* duplication occurring before or around teleost radiation. The syntenic analysis (Fig. 3) seemed to confirm this hypothesis showing that this two sequences *ffar2b2a* and *ffar2b2b* were included in the same synthenic group. Our synthenic analysis (Fig. 3) showed that the syntenic group *etfb-usf2-hamp-iglon5-ffar2b2a-ffar2b2b-ago1-ceacam6)* containing both *ffar2b2a* and *ffar2b2b* in trout on chromosome 2 was found in zebrafish on chromosome 16 containing one sequence of *ffar2b2b*, in Mexican tetra on chromosome 10 containing two sequence of *ffar2b2b* (*ffar2b2b*1, *ffar2b2b*2; data not shown) and in atlantic salmon on ssa05.

For the two rainbow trout *ffar2b2b* sequences*,* they grouped together (ENSOMYG000000030500 and ENSOMYG000000004604) in chromosome 2 and chromosome 3 respectively. These two sequences grouped together with two other teleosts sequences respectively (*i.e.* sequences ENSSAG00000052945 and ENSSAG00000068216 in salmon and ENSHHUG00000014406 and ENSHHUG00000022237 in huchen). These two *ffar2b2b* sequences for mexican tetra (ENSAMXG000000042613 and ENSAMXG000000043089) grouped together with one copie for zebrafish (ENSDARG000000079661). This tree configuration is in a favour of a *ffar2b2b* duplication occurring before or around salmonid radiation. Such hypothesis seemed to be confirmed by our synthenic analysis (Fig. 3) showing that the syntenic group *etfb-usf2-hamp-iglon5-ffar2b2b-ago1-dhdds* is found in trout on chromosome 2 and 3 surrounding *ffar2b2b1* and *ffar2b2b2,* in atlantic salmon on ssa05 and ssa02 (data not shown) and in huchen on QNTS01000300.1 and QNTS01001258.1 (only for *iglon5-ffar2b2b-ago1-dhdds* (data not shown).

### Phylogenetic analysis of *ffar3*

**Fig. 4:**
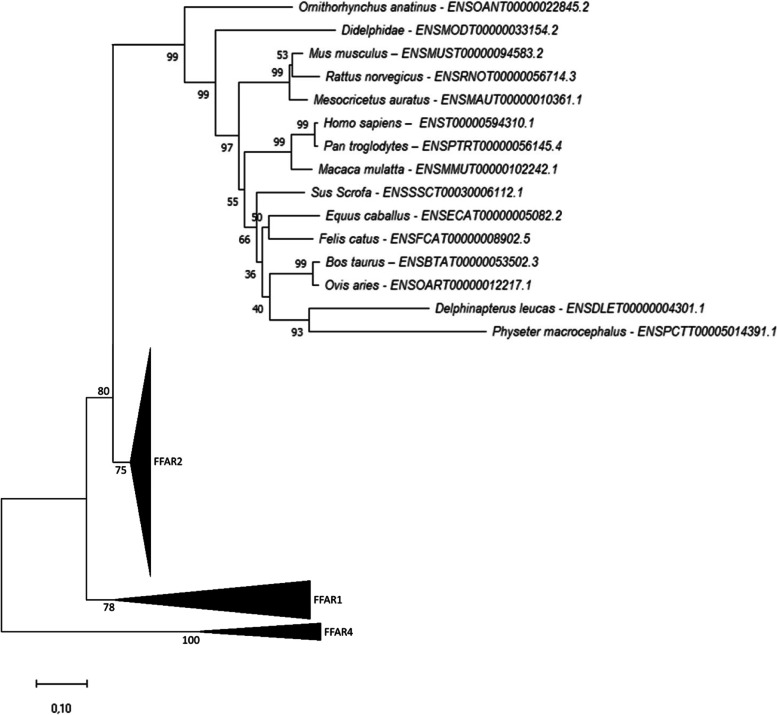
Phylogenetic tree of *ffar3* in vertebrates. FFAR3 protein sequences were aligned using the Maximum Likelihood Method (with Poisson model). Following alignment, the phylogenetic tree was constructed using the neighbour-joining method in Molecular Evolutionary Genetics Analysis (MEGA) software version 7.0 (Tamura 2013). The branch support values were gained by non-parametric bootstrapping (500 replicates). The scale bar represents the calculated evolutionary distance. Genbank accession numbers (from Ensembl or Genoscope databases) are specifed for each species

Through genome mining and phylogenetic analysis, we identified one gene related to the *ffar3* encoding gene in mammals including platypus, but no sequence was found in spotted gar nor in analysed teleosts (Fig. 4). Based on the automatic annotation provided in Ensembl, *ffar3* appeared to be lost in actinopterygii.

### Phylogenetic analysis of *ffar4*

**Fig. 5:**
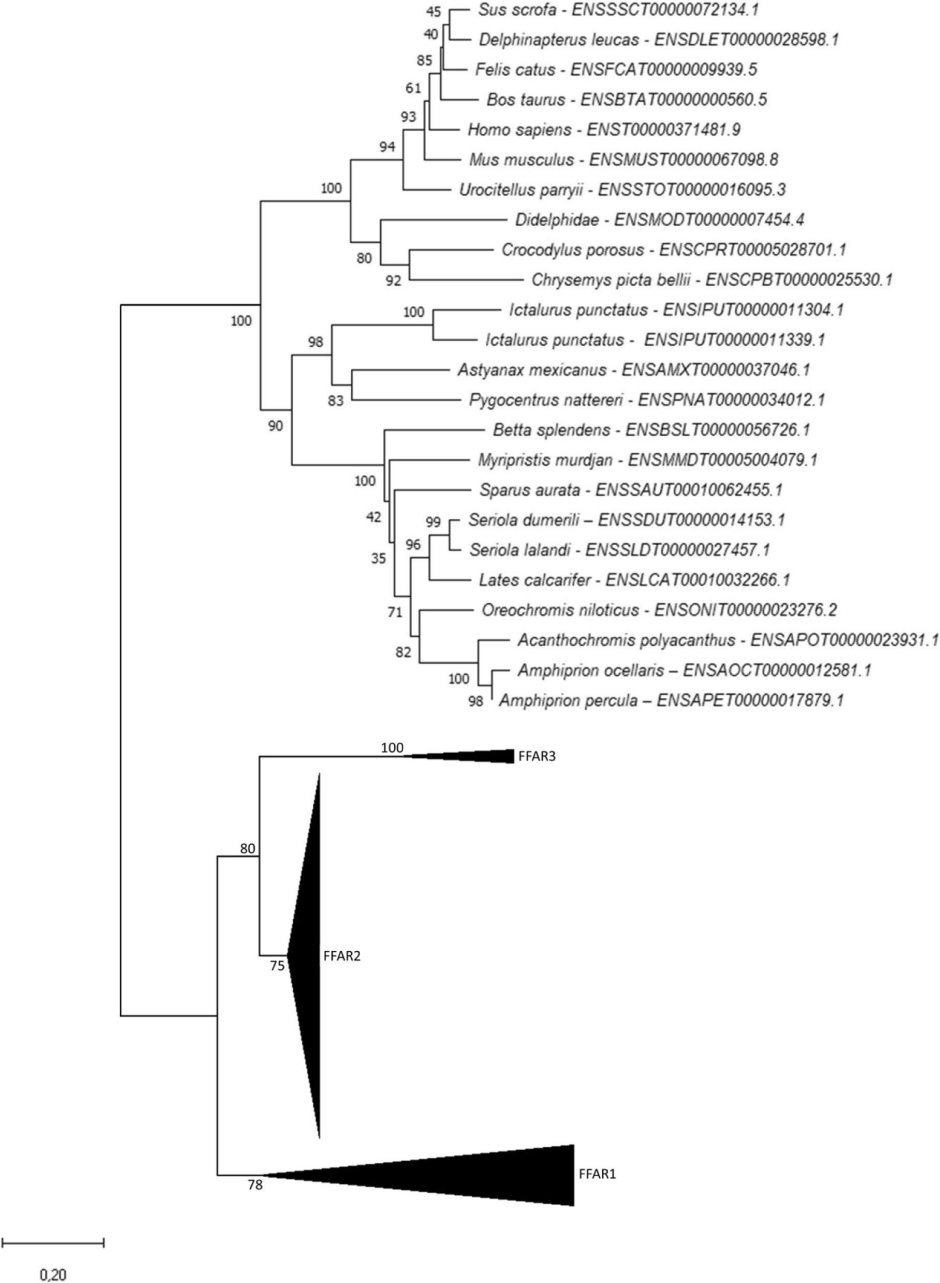
Phylogenetic tree of *ffar4* in vertebrates. FFAR4 protein sequences were aligned using the Maximum Likelihood Method (with Poisson model). Following alignment, the phylogenetic tree was constructed using the neighbour-joining method in Molecular Evolutionary Genetics Analysis (MEGA) software version 7.0 (Tamura 2013). The branch support values were gained by non-parametric bootstrapping (500 replicates). The scale bar represents the calculated evolutionary distance. Genbank accession numbers (from Ensembl or Genoscope databases) are specifed for each species

Through genome mining and phylogenetic analysis, we identified one gene related to the *ffar4* encoding gene in Sarcopterygii (Fig. 5). No sequence was found for spotted gar, and coelacanth. For teleost fish, we found no, one or two genes related to the *ffar4* encoding gene in characiphysae (none for zebrafish, one in astyanax or red-bellied piranha and two in channel catfish) depending on the species. For percomorphaceae, one gene was found for pinecone soldierfish, Eupercaria (gilthead seabream), carangaria (greater amberjack, yellowtail amberjack and barramundi perch), cichlidae (nile tilapia), and one for pomacentridae (spiny chromis, clow anemonefish and orange clownfish). No gene was found for salmonids fish. The Phylogenetic analysis grouped the different Ffar4 protein sequences of teleosts closest to mammalian FFAR4.

### mRNA levels of *ffar* genes during embryonic development.

**Fig. 6:**
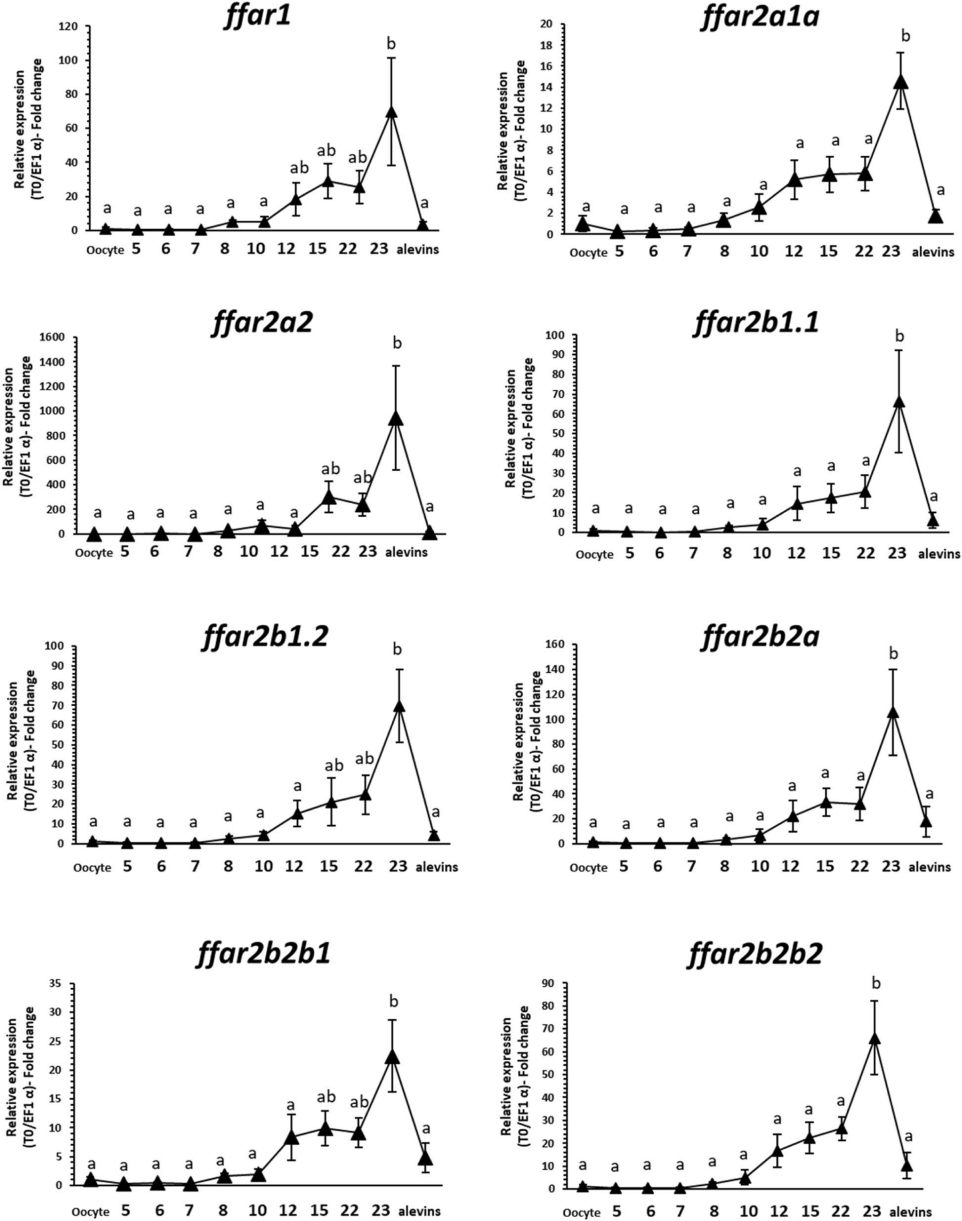
Expression pattern of *ffar1* and *ffar2* transcripts during development. Relative gene expression measured by RT-PCR of *ffar* genes family (*ffar1, ffar2a1, ffar2a2, ffar2b1.1 ffar2b1.2, ffar2b2a, ffar2b2b1, ffar2b2b2)* during ontogenesis. Embryos were sampled according to Vernier (1969) at stages oocyte (0), 5, 6, 7, 8, 10, 12, 15, 22 and 23, avelin (31). For all stages, gene expression level was normalized by the abundance of exogenous luciferase RNA. Data are expressed as means ± s.e.m. (*N* = 3 pools of embryos, one pool of 30 embryos per spawn). Different letters indicate significant differences between conditions (*P* < 0.05)

Real-time PCR was performed to determine the stage-specific mRNA levels of rainbow trout *ffar*-related genes during embryogenesis and in hatched alevins. This analysis showed (Fig. 6) that all mRNA sequences encoding *ffar genes* were detected and that mRNA levels increased after stage 10 to reach a maximum level at stages 23 (corresponding to the setting up of primitive organs) before to drastically decrease at alevin stage.

### mRNA levels of *ffar* genes after feeding response in gut and brain of rainbow trout fed by a challenge with plant based diet.

**Fig. 7:**
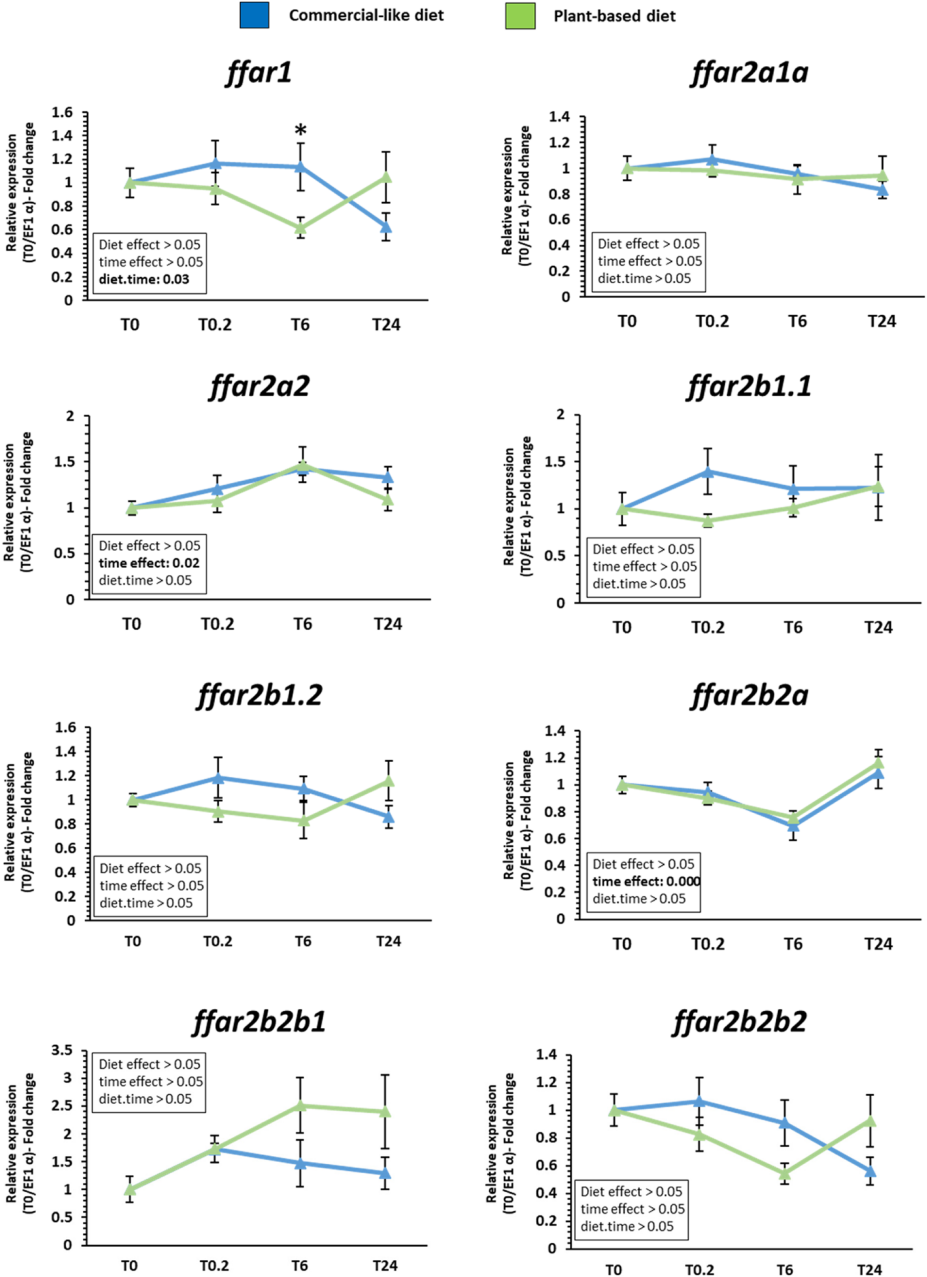
Expression pattern of *ffar1* and *ffar2* transcripts in proximal gut of rainbow trout fed with a commercial and plant-based diet following 5 fasted days. Relative gene expression measured by RT-PCR of *ffar* genes family (*ffar1, ffar2a1a, ffar2a2, ffar2b1.1 ffar2b1.2, ffar2b2a, ffar2b2b1, ffar2b2b2)* in proximal gut. Values are expressed as group mean ± SEM (*n* = 6); two-way ANOVA followed by Tukey post hoc test; if interaction (diet × time), letters indicate a significant difference between conditions as determined by a one-way ANOVA (*p* < 0.05)

**Fig. 8:**
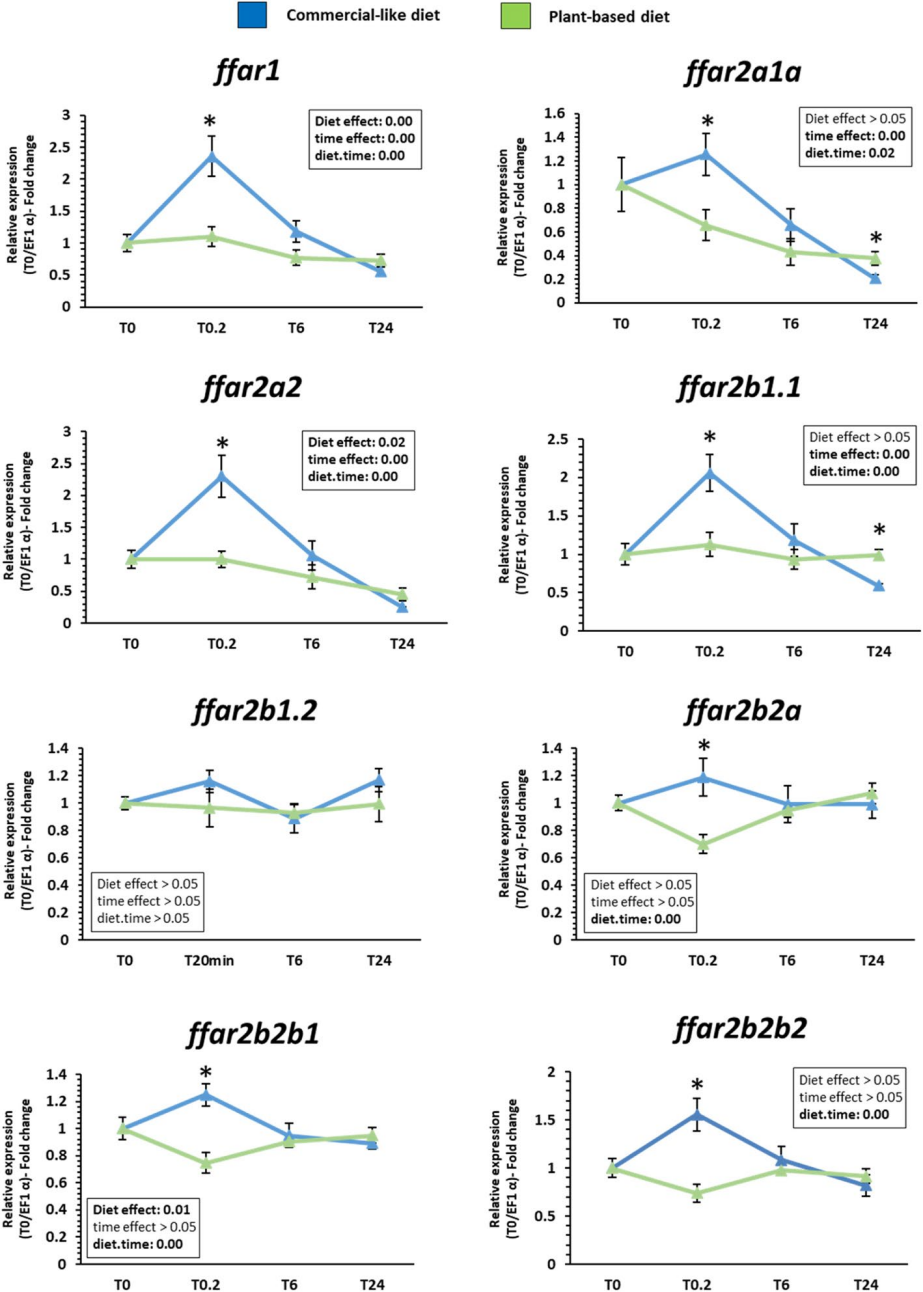
Expression pattern of *ffar1* and *ffar2* transcripts in brain of rainbow trout fed with a commercial and plant-based diet following 5 fasted days. Relative gene expression measured by RT-PCR of *ffar* genes family (*ffar1, ffar2a1a, ffar2a2, ffar2b1.1 ffar2b1.2, ffar2b2a, ffar2b2b1, ffar2b2b2)* in brain. Values are expressed as group mean ± SEM (*n* = 6); two-way ANOVA followed by Tukey post hoc test; if interaction (diet × time), letters indicate a significant difference between conditions as determined by a one-way ANOVA (*p* < 0.05)

After a single meal following five fasted days, mRNA levels of *ffar1* and *ffar2* in proximal gut and brain of rainbow trout are presented in Figs. 7 and 8 respectively. All mRNA sequences encoding *ffars* were detected.

In gut, mRNA level of *ffar1* was significantly affected by the interaction between the diet and the time after the meal with an increase in fish fed with commercial-like compared to those fed the plant-based diet (Fig. 7). Moreover, mRNA levels of *ffar2a2* and *ffar2b2a* were affected by the time after the meal but independently of the diet. The mRNA levels of *ffar2a1a, ffar2b1.1, ffar2b1.2, ffar2b2b1* and* ffar2b2b2* genes remained stable in time between fish fed with commercial-like and plant-based diet.

In brain, the mRNA levels of *ffar1* and* ffar2a2* were significantly affected by the diet, the time after the meal and the interaction between these two factors. *ffar1* and* ffar2a2* mRNA level were increased for fish fed with commercial-like *vs* plant-based diet 20 min after the meal (Fig. 8). The mRNA levels of *ffar2a1a* and *ffar2b1.1* were significantly affected by the time (increase and decrease respectively) after the meal, independantly of the diet but with the interaction between these two factors. *ffar2a1a* and *ffar2b1.1* mRNA level were increased for fish fed with commercial-like *vs* plant-based diet 20 min after the meal and decrease 24 h after the meal for same diet.

The mRNA levels of *ffar2b2a*, *ffar2b2b1* and *ffar2b2b2* were significantly affected by the interaction between the diet and the time after the meal (with diet effect for* ffar2b2b1*) with an increase of their mRNA level for fish fed with commercial-like *vs* plant-based diet 20 min after the meal. The mRNA levels of* ffar2b1.2* gene remained stable in time after the meal with commercial-like or plant-based diet.

## Discussion

In the present study, we deciphered a genomic overwiew of the *ffar* family in rainbow trout by phylogenetic and syntenic methods. We also integrated molecular measures to understand the temporal dynamics during development and in different nutritional conditions of the modulation of these *ffars* towards a better understanding of their functions in fatty acid regulation. Specifically, taking into consideration the complexity of the trout genome, our aim was to provide original understanding of *ffar genes* expression during ontogenesis, and to study the time course patterns of their genic regulation in gut and brain of trout fed with commercial or plant-based diet.

### Genomic overwiew of *ffar genes* in rainbow trout

Although only de-orphanised (discovery of ligands) in the recent past, FFARs responding to FFAs have attracted considerable attention in mammals [30]. Despite the importance of this family in numerous functions, there are still gaps in the knowledge of the teleost-specific FFARs related to evolution, nomenclature and function, which is exacerbated within the salmonids due to the SaGD (4R).

The recent availability of assembled genomes for rainbow trout provide an exhaustive research to establish the *ffar* genes repertoire and especially new coherent nomenclature of *ffar2* in rainbow trout. Hovewer, although well-assembled, the trout genome is not necessarily correctly annotated. This study has therefore carried out a deep phylogenetic and syntenic analysis in order to ensure also these annotations of the different sequences of *ffar* reported in the trout genome. This study identified no *ffar3* nor *ffar4,* one *ffar1* related gene and seven *ffar2* genes in trout. These first in silico analyses identified a wrong annotation of *ffar* sequences in trout where two *ffar2 (ffar2b1.1 ffar2b1.2)* sequences were initially annotated as *ffar3* (ENSOMYG00000041393 and ENSOMYG00000041387) and *ffar1* was not annotated in trout genome.

Phylogenetic analysis allowed to classify *ffar2* into two subclasses, that we named class a and b. Syntenic analysis showed a clear conservation of genomic organisation, further supporting the identity of these genes as *ffar2*.

For the first sub-tree, *ffar2a,* our phylogenetic analysis suggested that the duplication before or around the teleost radiation (TGD, into *ffar2a1* and *ffar2a2*) has resulted with a loss of *ffar2a* in non-salmonids fish but specie-specific with only one copie is found in zebrafish, mexican tetra and Medaka but two in nile tilapia. Moreover, the duplication before or around the salmonid radiation (SaGD; into *ffar2a1a*, *ffar2a1b and ffar2a2*) was followed by a loss of one *ffar2a2* sequence. This result is in accordance with Berthelot et al. findings who suggested that [20], when one loss of sequence is observed after the TGD, most of the time one loss is observed in SaGD, by a process termed gene fractionation [31]. Finally, our results suggested an additional loss in rainbow trout, with the presence of *ffar2a1b* sequence in huchen and atlantic salmon but not in trout.

Concerning *ffar2b1* sub-tree, our analysis suggested that the duplication before or around the teleost radiation has resulted with a tandem duplication for *ffar2b1* (into *ffar2b1.1* and *ffar2b1.2*) resulting of the duplication in salmonids. Here again, as suggested by Berthelot et al., [20], the genes retained in duplicated copies after the successive TGD events that occurred during vertebrate evolution were also more likely to be retained as duplicates following SaGD.

For *ffar2b2* sub-tree, our results supported the same conclusion than for *ffar2a* sub-tree with a duplication (into *ffar2b2a* and *ffar2b2b)* before or around the teleost radiation and with another duplication before or around the salmonid radiation. Finally, our results suggested an additional loss in rainbow trout for *ffar2b2a* with only one copie.

### mRNA levels of *ffar* genes increase at the formation of the primitive organs during embryo development

The early increase in mRNA levels of *ffar*-related genes (at stage 23, corresponding to the setting up of primitive organs) followed by an important decrease of these transcript at alevin stage could supported an important role of these receptors in the regulation of fatty acid. Indeed, this regulation would follow a massive production of energy (lipids) at the end of embryo stage. This could also reflect a phase of preparation for the catabolism of dietary nutrients at first feeding, as proposed for digestive enzymes like lipase [32] or previously observed in strickly same pattern of these results for duplicated glucose metabolism-related genes in rainbow trout [22]. Finally, our data may suggest an important role for each of these receptors at an early stage in rainbow trout, even before a role in the regulation of dietary fatty acids.

### Divergence patterns of mRNA levels expression of *ffar* genes in the fatty acid regulation in gut and brain of rainbow trout after nutritional challenge.

The conservation of duplicated *ffar2* genes in the trout genome offered an interesting model to study potential divergences in both the function and the expression of the related paralogues. Indeed, as suggested by Force et al., [33] the new duplicated genes can acquire new expression patterns potentially leading to neo- or subfunctionalization which could support the rise of new molecular and cellular functions, and can play an important role in phenotypic variability but could also be either silenced.

FFARs (class 1, 2, 3 and 4) are known to be responsible in part for the various biological and physiological functions of FFAs through their binding to these receptors [34]. Indeed, FFAs are not only an essential energy source, but also function as signaling molecules that regulate various cellular processes and physiological functions according to carbon chain length via FFARs activation through their binding. In mammals, FFARs are expressed in various tissues and influenced many important metabolic functions that maintain energy homeostasis [35]. Moreover, the FFARs-mediated signaling transduction pathways in the regulation of metabolism in intestinal tract is well known as well as the biological role (energy balance, immune responses, fat preference…) of the FFAs via the activation of the FFARs in central nervous system [6]. Our phylogenetic analysis revealed that *ffar3* and *ffar4* were not present in rainbow trout genome. Knowing the important role of these two FFARs receptor in mammals especially their implication in various biological and physiological functions such as energy regulation, immunological homeostasis, and neuronal functions to the regulation of energy homeostasis [36, 37], this is a surprising finding. However, all FFARs are known to have similar roles even thought the fatty acids (short *vs* medium or long chain) that bind to it differ. Thus, we could hypothesise that the *ffar1* and the seven *ffar2* paralogues could assumed the functions of the lost *ffar3* and *ffar4* reported in others species.

Firstly, the detection of all *ffar* transcripts in gut and brain tissues of rainbow trout could assumed that *ffar1* and *ffar2* could have an important role in the digestive tract and central nervous system in rainbow trout. Especially, their modulation by diet changes could assume a role in the regulation of fatty acid that varies according to the diet composition. Considering that FFAR1 are known to be activated by ω-3 LC-PUFA, while FFAR2 are activated by SCFAs in mammals [6], based on our results, it could be also the case in rainbow trout. Indeed, a unique meal of plant-based diet totally devoid of LC-FFAs decreased *ffar1* mRNA level 20 min and 6 h after the meal respectively in brain and gut and 20 min for all *ffar2* mRNA level except for *ffar2b1.2* in brain of trout*.* Even if no study has been done on teleost fish, these results were consistent with the time course expression of others receptor genes (peroxisome proliferator activated receptor) involved in fatty acid metabolism [38]. Authors concluded that FFAs were able to rapidly induce (less than 1 h with higher expression to 6 h) the expression in muscle cells of mice of key genes involved in their catabolism and that the LC-PUFAs mixture had a positive role increasing the expression of master metabolic regulators and their downstream target gene. Furthermore, Mobraten et al., revealed that LC-PUFAs enhanced the cytosolic concentration of the signaling pathways of FFARs (calcium and MAP kinase ERK1/2) with the same efficiency, but with different kinetics (on average 20 min) and intensity in muscle cells [39]. FFAR1 is also reported to be specifically activated by LC-PUFA (DHA) in primary cortical neurons of mouse model, significantly alleviated cognitive functions in mice. This effect was mediated by an increase of intracellular calcium less than 20 min and by the extracellular receptor kinase (ERK) and P38-mitogen-activated protein kinase (MAPK) pathways after FFAR1 activation [40]. All these finding support our present results suggesting that the biological role of LC-PUFAs could be depending in rainbow trout by their binding to the FFAR family then their rapid modulation (than 20 min).

About gene function, if we assume that as in mammals the activation of FFAR is associated with increase of genic expression, we can make some assumption to their potential function in trout based on the results. Our results were in agreement with other studies [41], namely an increase of the genic expression of *ffar4* (GPR120) just after meal, which could regulate the postprandial mechanisms of fat eating behavior. The increase of *ffars* expression can indicated an activation of receptors, the FAs released in the mouth cavity activated receptors in brain tissues and after 6 h, the expression of receptors return to a basal condition. The literature shows us that, the early activation of FFAR2 could have a role in the regulation of appetite through a variety of mechanisms related to its activation [42]. Finally, numerous studies concluded that FFAR1 and FFAR4, could plays a critical role in various physiologic homeostasis mechanisms especially the regulation of appetite, eating disorder, or food preference [6, 43, 44]. Moreover, it is known that the activation of FFAR is related to gene expression of *ffars* increased after a meal. Ozdener et al., demonstrated that, in humans and mice, an incubation of taste bud cells with an ω-3 LC-PUFA (ALA) induced an upregulation of *ffar4* [45]. A report of Choo et al. (2020), which studied the effect of maternal obesity on the expression of receptors in the offspring, observed an increase of *ffar4* expression of female offspring of high-fat diet (HFD) fed mice [46]. These studies are in agreement with our results, which could suggest an activation of FFAR lead to differential physiologic homeostasis mechanisms in trout fed with C diet especially in the regulation of appetite, eating disorder, or food preference. FFAR receptors could have a preponderant role in the growth performance throughout the life cycle of the rainbow trout.

Moreover, these finding demonstrated that gene duplication events of *ffar2* offered an interesting model to study potential divergences in both the function and the expression of the related paralogues. Thus, the expression pattern between all *ffar2* paralogues were not different in gut tissue where as discrepancies in responses are observed in the brain. In fact, if we consider that all *ffar2* (except *ffar2a2*) were differentially regulated by the diet and specifically at 20 min with up-regulation for trout fed commercial-like diet, many divergent patterns between paralogues were observed. Interestingly for *ffar2a* genes*, ffar2a2* and *ffar2a1a* genes displayed the same expression pattern. For *ffar2b* genes, the two paralogues *ffar2b2b1*, *ffar2b2b2* and *ffar2b2a* genes displayed the same expression pattern where as the tandem duplicated *ffar2b1.1 and ffar2b2.2* genes had divergences of their expression after a meal. This finding demonstrated that even if genes are duplicated and therefore very close at the phylogenetic and syntenic level, the modulation of their expression is complex and most of the time results from the combination of different mechanisms. Indeed, gene duplications provide an essential source of genetic redundancy but does not necessarily tenfold the function of the gene. In salmonids, loss of gene functionality is slow, with only 50% of genes loosing function after 50 million years [47] and these duplicated genes may instead undergo neo- or sub-functionalization [33]. All these results here confirmed that *ffar2* paralogues in rainbow trout were sub- or neo-functionalized as they were differentially regulated by nutritional statut and/or by the meal.

## Conclusion

Overall, for the first time in rainbow trout, through genome mining and phylogenetic analysis, we identified and characterised 7 coding sequences for *ffar2* in salmonid species where as no *ffar3* and *ffar4* gene have been reported. The differential expression of trout *ffar2* genes identified here by nutritional status or feeding may therefore provide evidence of the varying functions of these duplicated genes in rainbow trout. The quantitative assays designed here for individual *ffar* genes will improve our ability to conduct expression studies as they allow for a more precise characterization of expression and can be utilized to unravel the potential contribution of individual *ffar* genes in rainbow trout in their various functions. For example, further studies will be necessary to characterize the potential binding (agonist/antagonist) and the role of these individual receptors in the detection and regulation of FFA sensing, metabolism and role in rainbow trout and to elucidate their implication in the regulation of feeding behavior. This knowledge will be important in the aquaculture industry for diversification or substitution of feed ingredients, especially the already expensive and limited FM/FO.

## Supplementary Information

**Additional file 1. **Normalized and raw RT-PCR datas in all analyzed tissus and conditions.

**Additional file 2: Supplemental information.** Protein sequence encoded by FFAR.

## Data Availability

All data generated or analyzed during this study are included in this published article, its supplementary information file (RT-qPCR datas), and online (DNA and protein sequences; all obtained from Ensembl databases; http://www.ensembl.org, Ensembl Release 105; November 2021; *eef1a1:* ENSOMYG00000038328*ffar1*: ENSOMYG00000041396; *ffar2a1a*: ENSOMYG00000004986; *ffar2a2:* ENSOMYG00000030315; *ffar2b1.1*: ENSOMYG00000041393; *ffar2b1.2*: ENSOMYG00000041387; *ffar2b2a*: ENSOMYG00000030493; *ffar2b2b1:* ENSOMYG00000030500; *ffar2b2b2*: ENSOMYG00000005604).
