# Supplementary material for: Characterization of free fatty acid receptor family in rainbow trout (Oncorhynchus mykiss): towards a better understanding of their involvement in fatty acid signalisation
Source: BMC Genomics. 2023 Mar 20;24:130. doi: 10.1186/s12864-023-09181-z (PMC10029227; doi:10.1186/s12864-023-09181-z)
Supplement: Supplementary file 2 — Additional file 2: Supplemental information. Protein sequence encoded by FFAR. [file 12864_2023_9181_MOESM2_ESM.docx]

Supplemental information. Protein sequence encoded by FFAR.

*ffar1*: [ENSOMYG00000041396](http://www.ensembl.org/Oncorhynchus_mykiss/Gene/Sequence?db=core;g=ENSOMYG00000041396;r=18:35017375-35018421;t=ENSOMYT00000097759)

MQVPVKDCVSLTVYSFTFLLGLPSNLLVLFVYVRKARKRGATPNVVYALNLCLANLALMAWLPVKALETFLQDWALPSPLCPVYSFFLFSSMYGSCLFLTAVTVGRYLSIAFPISYKLYRRGRISCFISAALWAVVLLHLSLGLVAEGGGGFVSTSSHNVSVCFENFTQDQLDLLLPLRLEMALLLFLMPLAITAFCTLRCVALVWRSCLPVLGKRRVLAVALSTLAVFVVCYVPYNASHIVGFVLQENVHWRTEAMLSSACNVFLEPVVMLMLSPATPRGLMGRLCGRPSRYSRTEGRHCSKTITRDPLANVRGVASLTDRQTGANISKLCQEKSAQTRHIRPVSGI

*ffar2a1a* : [ENSOMYG00000004986](http://www.ensembl.org/Oncorhynchus_mykiss/Gene/Sequence?db=core;g=ENSOMYG00000004986;r=3:27202882-27204851;t=ENSOMYT00000010991)

SQSGPHTILTTKLSPGFNCEVILSVYIITFLIGLPANILALYAFSIKIHKKPTPTDILLLNLIVSDLLFLLFLPLKMYEAASGMQWYLPEFLCSITSYTFFSTIYTSSLLLMAVSVVRYLAVAFPITYRQLSKPFYSVVSSTIIWLLTTAHCSIVFIIQHHQDLSQSNTSVCYENFTKQQLTILLPVRLEFFVMLCLVPLMVCIFCYLRCIWILYMLPRISPGQKQKAIGMALGTLAVFLVCVFPYNFSHVLGFLTGHSPPWRYYTLLLSAFNTCLDPIIFYFSSSTFRITIKMSICKMLGLRHGQAAIQRESTIADIGQE

*ffar2a2 :* [ENSOMYG00000030315](http://www.ensembl.org/Oncorhynchus_mykiss/Gene/Sequence?db=core;g=ENSOMYG00000030315;r=2:35786777-35788108;t=ENSOMYT00000071257)

MTVHYCYLSLGPDSALGVILSVYIITFLVGLPGNILALYAFSVKIHNKPTPTDILLLNLTVSDLIFLLFLPLKMHEAASGMVWTLPRLLCNVTSFVFFSTIYTSSLLLMVVSVDRYLCVAFPVQYRLRRKPLYGVVSSLVVWVFSSVHLCFIYIVENQTSSDLFTCYNNFTQEQLKVVLPMRLELCVVLYIVPLLVCVFCYLNFILILNRTPNLCAEKRKRAVGMAVGTLLVFVVCFLPYNVTHVQGFIIQDNVEWRLYALLLTTVNTVLDPVTFYFSSSMFQRRNSTPEGHCNPATTEGGLSDK

*ffar2b1.1* : [ENSOMYG00000041393](http://www.ensembl.org/Oncorhynchus_mykiss/Gene/Sequence?db=core;g=ENSOMYG00000041393;r=18:35012485-35016128;t=ENSOMYT00000097752)

MVTSEEKAFFVTTTPTVSANMRPANDISCSFALFVYITTFLIGVPANILAFCTFCRKVRRKPAPIDILLLNLTISDLIFLAFLPFKMKEAVDDFNWTLPYFLCPVTGYLFFSTIYNSTLLLTAVSVERFLSVAYPVRFTAPGRVFHTQLACAVFWILSLAHCSVVFVMQLDKDADNTTCYGNFNDVQLITLLPVRLEMSLVLFCVPFLISTFCYVNFIRILSRLPNISQHRRLRAIGLALGTLLVFALCFGPYNVSHMVGIMQNKVPTWRNNAILLTTLNACLDPIVFYFSSSAVRSTLSLCLKRIKAQLNPKATAAHAGNTPDPMDSTHSSSQKFVAAAKSGTNYTPR

*ffar2b1.2* : [ENSOMYG00000041387](http://www.ensembl.org/Oncorhynchus_mykiss/Gene/Sequence?db=core;g=ENSOMYG00000041387;r=18:34997306-34998794;t=ENSOMYT00000097744)

MPITHLLLSVYIATFLIGVPANILAFCTFCQKVRRKPAPIDILLLNLTISDLIFLTFLPFKMKEAVDDMHWNLPFFLCPVTGFLFYSTIYNSTLLLTAVSVERYLGVAFPIRYALCHRPRYAVVASIICWVVSSLNLSVVYIVPCSHWIYSNGTIMDDPPTTCYLNFTQGQLSILLPVRLELFLVLFCVPFLVCTFCYVNFIRILSRLPNIGRRRRLRAIGLALGTLLVFALCFGPYNVSHVVGFVRKDSESWRDVALLSSTLNACLDPIIFYFSSVAVRSMLRTVNKSTTALFCAGLDKYR

*ffar2b2a* : [ENSOMYG00000030493](http://www.ensembl.org/Oncorhynchus_mykiss/Gene/Sequence?db=core;g=ENSOMYG00000030493;r=2:36075038-36076087;t=ENSOMYT00000071657)

MQECHTALCLSVYLVTFLTGLPANAVAFYTFSKKVRQKPTPIDILLLNLTISDLLFLLFLPFKMQEVTDDMTWSLPYILCPLSGFFFYMTIYVSTLFLTAVSVERYLGVAFPIQHSLKRRPLYAVVASVFIWVFSILHLSIVVIMPYYNPPQDSLSSTTNSSNIYEFSNVSNVLISDSDNIVSSRNVCYEDFSKKQLAILLPVRLELCLVLFCVPFLICSFCYINFIRILSGLPHIGRRRRLRAIGLALGTLLVFAFCFGPYNVSHIVGFITRKNPDWRDMALLCSTFNACLDPFIFYFSSSAVRGTLGSMLQGARIKLAKCHIHWSPWKGMSEPPIDKGPKQAEMNAI

*ffar2b2b1 :* [ENSOMYG00000030500](http://www.ensembl.org/Oncorhynchus_mykiss/Gene/Sequence?db=core;g=ENSOMYG00000030500;r=2:36083966-36085089;t=ENSOMYT00000071674)

QPSCEIFLVLVVYIITLLIGFPANVVAFYTFSKKVRQKAMPVDILLLNLTISDLIFLLFLPFKIKEVADNMKWIMPHFLCPLTSFVFYTTIYNSTFFLTAISVERYLGVAFPIKYKLKRRPLYAMVASVFFWAISMAHISIVYIIQYFDYSNTTRADPLNLDMCYEDFTPEQLQVLIPVRLELFLVLFCVPFFICCFCYINFIRILSQLPSINRKKRQRAIGLSLGTLLVFIVCFAPYNLSHVVGFVNWESPTWRVEALLSSTVNASLDPIIFYFSSAALRSTFHLFMKNLVERMQRLFFCNKVLFCSRTQKDSTPSTNDSSL

*ffar2b2b2* : [ENSOMYG00000005604](http://www.ensembl.org/Oncorhynchus_mykiss/Gene/Summary?db=core;g=ENSOMYG00000005604;r=3:27543771-27544829;t=ENSOMYT00000012347)

MLSGNESGDRHSHSSLVLMVYIITFLIGLPANGVAFYTFGKKVRQKAMPIDILLLNLTVSDLLFLLFLPFKMKEAADDNVWNMPVFLCPLTSFVFYATIYNSTFFLTAISVERYLGVAFPIKYKLIRRSLYTTVASVAFWVISMAHVSIVYIIQYLVDSSATQEGDMCYTNFTQMQLQVLIPVRLELFLVLFCVPFFICCFCYINFIRILSQLPSINPKKRLRAIGLSLATLLVFIVCFAPYNLSHLVGFVNWESPNWRMAALLSSTINASLDPIIFYFSSSALRCTFHLFLKNLLESVQGLCFCSKALYCPVLFCTRTQKDSTPSSNDNMCQTHSTEGQGSAGFRSTGVLD
